# Flaxseed Oil Supplementation Augments Antioxidant Capacity and Alleviates Oxidative Stress: A Systematic Review and Meta-Analysis of Randomized Controlled Trials

**DOI:** 10.1155/2021/4438613

**Published:** 2021-09-03

**Authors:** Vali Musazadeh, Jaber Jafarzadeh, Majid Keramati, Meysam Zarezadeh, Mahshid Ahmadi, Zohreh Farrokhian, Alireza Ostadrahimi

**Affiliations:** ^1^Student Research Committee, Tabriz University of Medical Sciences, Tabriz, Iran; ^2^School of Nutrition and Food Sciences, Tabriz University of Medical Sciences, Tabriz, Iran; ^3^Nutrition Research Center, School of Nutrition and Food Sciences, Tabriz University of Medical Sciences, Tabriz, Iran; ^4^Department of Nutrition, Marand Branch, Islamic Azad University, Marand, Iran

## Abstract

**Objective:**

Studies have reported controversial findings regarding the flaxseed oil effect on antioxidant status biomarkers. The present meta-analysis aimed to determine the impact of flaxseed oil on the serum level of biomarkers of oxidative stress.

**Methods:**

A systematic search was conducted up to November 2020 on PubMed, Embase, Web of Science, Scopus, and Cochrane Central Library. Random-effects model was employed to perform meta-analysis. Subgroup analysis was carried out to determine the effect across different ranges of dosages and durations.

**Results:**

Eight trials were included with a total sample size of 429 individuals with a mean age range of 25 to 70 years. The results indicated that flaxseed oil supplementation led to a significant decrease in malondialdehyde (MDA) levels (SMD: −0.52 *μ*mol/L; 95% CI: −0.89, −0.15; *P*=0.006, *I*^2^ = 71.3, *P* < 0.001) and increase in total antioxidant capacity (TAC) levels (WMD: 82.84 mmol/L; 95% CI: 19.80, 145.87; *P*=0.006, *I*^2^ = 92.7, *P* < 0.001). No significant effect was observed on glutathione (GSH).

**Conclusion:**

Our findings revealed that flaxseed oil supplementation might play a beneficial role in the reinforcement of the antioxidant defense system and amelioration of oxidative stress in adults.

## 1. Introduction

Oxidative stress (OS) is an imperfection in the balance between reactive oxygen/nitrogen species (ROS/RNS) and the total antioxidant capacity of organisms [1]. An increase in the oxidation of the biomolecules and dysfunction of the antioxidant system subscribe the pathogenesis of the chronic disease [2] including atherosclerosis, type 2 diabetes (T2DM), fatty liver diseases, hypertension, and neurological diseases [3–6]. Different markers have been used in studies to evaluate this balance between ROS/RNS production and antioxidant system function such as total antioxidant capacity (TAC), malondialdehyde (MDA), and glutathione (GSH). MDA, as the main determinant of the oxidation status in the body, is a by-product in the lipid peroxidation pathway. On the contrary, TAC and GSH are the markers of the antioxidant defense system [7]. The TAC consists of several enzymes and biomolecules (such as GSH, superoxide dismutase (SOD), glutathione peroxidase (GPx), and catalase (CAT)) that, individually or in combination with each other, play a role in modulating redox reactions [8]. In fact, glutathione, as a part of TAC, is a cysteine-containing peptide involving in the antioxidant system via oxidation and reduction in the thiol domain [9]. Dietary pattern and nutrition have been identified as important factors of oxidative stress [10]. Previous data have revealed that flaxseed oil might have antioxidant properties and ameliorate oxidative stress [11].

Flaxseed oil is a rich source of alpha-linolenic acid and some effective phytochemicals such as lignan (secoisolariciresinol diglucoside-SDG), phenolic acids, and flavonoids [12–14]. It has many properties including antioxidant [15], antiatherosclerotic [16], and anti-inflammatory [17]. Flaxseed oil has been shown to enhance the function of the antioxidant system due to its antioxidant compounds such as phenols and vitamin E. It also reduces blood lipids and inflammation by increasing LDL receptors leading to reducing MDA either [18, 19]. However, the results of studies about the effect of flaxseed oil on oxidative stress parameters are conflicting [20]. Some clinical trials have reported that flaxseed oil significantly decreases serum concentrations of MDA and increases TAC and GSH [21–24], while there were some studies that did not find any significant effect [20, 25]. A previous meta-analysis published in 2020 assessed the antioxidant effects of flaxseed oil supplementation on biomarkers of oxidative stress using 5 studies. The results showed that flaxseed oil reduces MDA and increases TAC levels, but no significant effect was observed on GSH levels [26], although changes of some biomarkers of oxidative stress have not been assayed in detail. Therefore, present meta-analysis was conducted to summarize current evidence and estimate the direction and magnitude of the effects of flaxseed oil supplementation on oxidative stress biomarkers in adults.

## 2. Materials and Methods

### 2.1. Search Strategy

The scientific international databases, including PubMed, Embase, Web of Science, Scopus, and Cochrane Central Library, were searched for relevant studies published up to November 2020. In the search strategy, MeSH terms and keywords were included without using language limitations (except Persian language). The search was conducted using the following search pattern: [(“Flax” [MeSH])OR (“flaxseed” [tiab]) OR (“flaxseed” [tiab]) OR (“linseed”[tiab]) OR (“lignan” [tiab]) OR (“whole flaxseed” [tiab]) OR (“ground flaxseed” [tiab]) OR (“flaxseed oil” [tiab]) OR (“Linum usitatissimum” [tiab]) AND [(“Oxidative Stress” [MeSH) OR (“Oxidative Stress” [tiab]) OR (“Total Antioxidant Capacity” [tiab]) OR (“anti-oxidant” [tiab]) OR (“Oxidant” [tiab]) OR (“reactive oxygen species” [tiab]) OR (“Malondialdehyde” [tiab])OR(“glutathione” [tiab]) OR (“TAC” [Title/Abstract]) OR (“GSH”[tiab]) OR (“MDA” [tiab])] AND [(“InterventionStudies” [tiab) OR (“intervention” [tiab) OR (“controlledtrial” [tiab]) OR (“randomized” [tiab]) OR (“randomised” [tiab]) OR (“random” [tiab]) OR (“randomly” [tiab]) OR (“placebo” [tiab]) OR (“assignment” [tiab]) OR (“randomized controlled trial” [tiab]) OR (“randomized clinical trial” [tiab]) OR (“RCT” [tiab]) OR (“double blinded” [tiab]) OR (“trial” [tiab]) OR (“controlled clinical trial” [tiab])]. The wild-card term “^*∗*^” was used to increase the sensitivity of our search strategy.

### 2.2. Inclusion and Exclusion Criteria

RCTs with the following criteria were included in our meta-analysis: either crossover or parallel design, investigating the effect of flaxseed oil on oxidative stress parameters and providing sufficient data on oxidative stress parameters (MDA, GSH, and TAC) at baseline and the end point for both intervention and control groups. Other studies, including in vitro, in vivo, and ex vivo studies, observational studies (cross-sectional, case-control, and cohort), and quasi-experimental studies, were excluded from this meta-analysis. Besides, studies on pregnant and lactating women were excluded.

### 2.3. Data Extraction

Two independent reviewers (MA and ZF) screened the articles according to the inclusion criteria. The title and abstract of the studies were reviewed in the first step. Then, relevant studies were evaluated to ensure the suitability of a study for full-text assessment. Any disagreement was resolved by discussion with the senior author (AO).

From the selected studies, the following data were extracted and entered in the review: first authors' name, publication year, sample size, study location, mean age of participants, the dose of flaxseed oil, study design, study duration, and baseline and end-point values (as means and SDs) for MDA, GSH, and TAC in both intervention and control groups.

### 2.4. Risk of Bias Assessment

The Cochrane Collaboration's risk of bias tool was employed to assess the risk of bias for each study. The tool consists of seven domains, including random sequence generation, allocation concealment, performance bias, reporting bias, detection bias, attrition bias, and other sources of biases. If the study contains a methodological defect, each domain was given a “high-risk” score that may affect its findings; if there was no defect for that domain, a “low-risk” score was given; if the information was insufficient to determine the effect, an “uncertain risk” score was given. If the trial had “low risk” for all domains, a high-quality study was considered a totally low risk of bias. Risk bias assessments were performed independently by two reviewers [27, 28].

### 2.5. Data Synthesis and Statistical Analysis

To obtain the overall effect size, mean and SD changes in the flaxseed oil and control groups were analyzed. When mean and SD changes were not reported in the included studies, the values were calculated by subtracting baseline values from end-point values. Medians, standard errors (SEs), confidence intervals (CIs), and interquartile ranges (IQRs) were transformed into means and SDs using the method of Hozo et al. [29]. We applied a random-effects model to obtain the overall effect size if the amount of between-study heterogeneity was significant. Heterogeneity was determined using *I*^2^ statistics and Cochrane's *Q*-test. *I*^2^ value > 50% or *P* < 0.1 for the *Q*-test was considered as significant heterogeneity [30, 31]. If the amount of heterogeneity was not significant, fixed-effects model was employed to estimate the overall effect size. To discover possible sources of heterogeneity, subgroup analyses were performed according to the predefined variables including duration of the intervention (≥8/<8 weeks), flaxseed oil dosage (≤2000/>2000 mg), sample size (*n* ≤ 26/*n* > 26), mean age of participants (≤48/>48 years), and the type of flaxseed products (flaxseed oil/omega-3 fatty acid from flaxseed oil). The sensitivity analysis was used to determine the dependence of overall effect size on a particular study. Egger's test and visual inspection of the funnel plot were used for identifying publication bias. In case of the presence of publication bias, trim-and-fill analysis was performed to simulate a model without publication bias by presenting a new effect size with inserting new fictitious studies. All the statistical analyses were performed using Stata, version 16 (Stata Corporation, College Station, TX, US). *P* < 0.05 was considered as the significance level.

## 3. Results

### 3.1. Systematic Review

**Figure 1:**
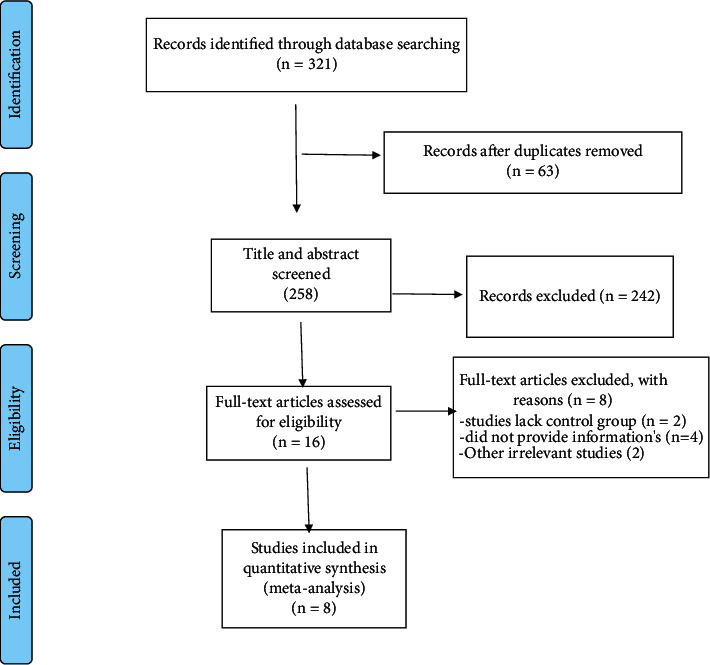
Flow diagram of study selection.

Eight effect sizes from 8 RCTs published between 2011 and 2020 were included in the systematic review and meta-analysis. The flow diagram of the study selection procedure is presented in Figure 1. Overall, 429 participants, including 216 participants in the intervention group and 213 participants in the control group, were enrolled in the review. The general characteristics of the studies are included in Table 1. Eight studies were conducted in Iran [11, 20, 23, 24, 32–34] and 1 in Brazil [35]. Participants of these studies had gestational diabetes mellitus [11], diabetic nephropathy [23], nonalcoholic fatty liver disease [34], metabolic syndrome [33, 35], diabetic foot ulcer [32], type 2 diabetes with coronary heart disease [24], and hemodialysis [20]. The average age of participants was 53 years. Flaxseed oil alone or plus with omega-3 fatty acids extracted from flaxseed oil cosupplementation was used as a treatment in the RCTs included. The duration of the intervention varied from 4 weeks to 12 weeks among studies.

### 3.2. Risk of Bias Assessment

In all trials, except the study conducted by Pilar et al. [35], participants were randomly assigned to the intervention and control groups, and eight studies described a random sequence generation method. The blinding of subjects and researchers was reported in six studies. Five studies [11, 23, 24, 32, 34] were judged to be of high quality. The results of evaluating the quality of the included studies are presented in Table 2.

### 3.3. Meta-Analysis

#### 3.3.1. Effects of Flaxseed Oil on MDA Levels

**Figure 2:**
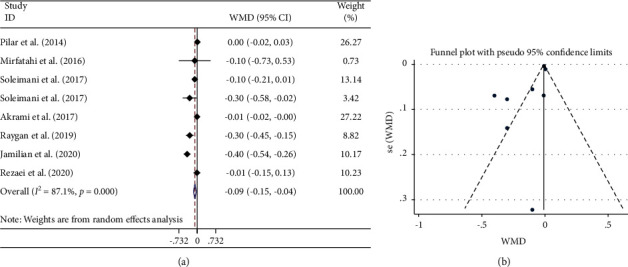
Forest plot (a) detailing standardized mean difference and 95% confidence intervals (CIs) and funnel plot (b) displaying publication bias in the studies reporting the effects of flaxseed oil supplementation on serum MDA concentrations. Horizontal lines represent 95% CIs. Diamonds represent pooled estimates from random-effects analysis. MDA: malondialdehyde; SMD: standardized mean difference; CI: confidence interval.

A pooled analysis of 8 RCTs on MDA levels showed a significant decrease following the intervention (SMD: −0.52 *μ*mol/L; 95% CI: −0.89, −0.15; *P*=0.006) (Figure 2(a) and Table 3). The level of heterogeneity was considerable (*I*^2^ = 71.3%, *P* < 0.001), in which mean age, treatment dosage, sample size, and duration of studies were shown as sources of it (Table 3). There was no significant change in results after subgroup analysis by mean age, intervention duration (≤8), dosage (≤2000 mg), study sample size, and type of flax products (flaxseed oil/omega-3 fatty acid from flaxseed oil) (Table 3).

#### 3.3.2. Effects of Flaxseed Oil on GSH Levels

**Figure 3:**
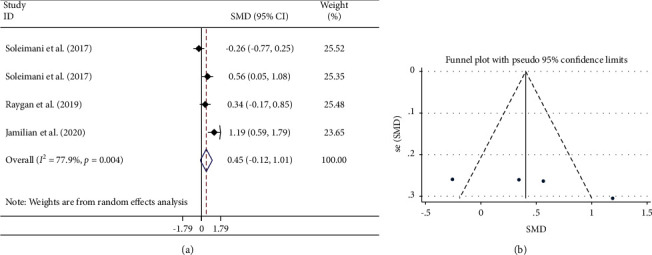
Forest plot (a) detailing standardized mean difference and 95% confidence intervals (CIs) and funnel plot (b) displaying publication bias in the studies reporting the effects of flaxseed oil supplementation on serum GSH concentrations. Horizontal lines represent 95% CIs. Diamonds represent pooled estimates from random-effects analysis. GSH: glutathione; SMD: standardized mean difference; CI: confidence interval.

Combining the data of 4 studies showed no significant effect on GSH levels (SMD: 0.45 *μ*mol/L; 95% CI: −0.12, 1.01; *P*=0.122) (Figure 3(a) and Table 3). There was a significant between-study heterogeneity (*I*^2^ = 77.9%, *P*=0.004) in which the mean age of individuals was identified as the source of high heterogeneity following subgroup analysis (Table 3). However, significant effects were observed in participants with a mean age of ≤48 years (SMD: 1.19; 95% CI: 0.59, 1.79) and flaxseed-derived omega-3 fatty acid subgroup (SMD: 0.43; 95% CI: 0.12, 0.74) (Table 3).

#### 3.3.3. Effects of Flaxseed Oil on TAC Levels

**Figure 4:**
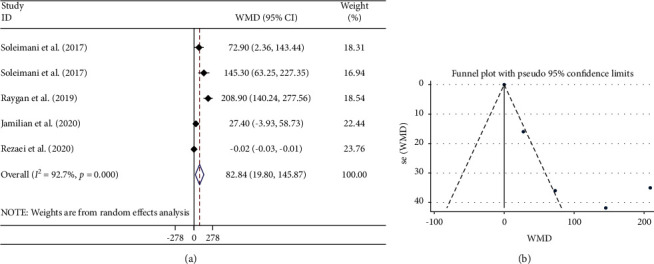
Forest plot (a) detailing weighted mean difference and 95% confidence intervals (CIs) and funnel plot (b) displaying publication bias in the studies reporting the effects of flaxseed oil supplementation on serum TAC concentrations. Horizontal lines represent 95% CIs. Diamonds represent pooled estimates from random-effects analysis. TAC: total antioxidant capacity; WMD: weighted mean difference; CI: confidence interval.

A significant increase in the TAC level was observed by flaxseed oil supplementation in the combined analysis of 5 studies (WMD: 82.84 mmol/L; 95% CI: 19.80, 145.87; *P*=0.01) (Figure 4(a) and Table 3). Significant between-study heterogeneity (*I*^2^ = 92.7%, *P* < 0.001) decreased by subgrouping subjects by their mean age and flaxseed type.

#### 3.3.4. Sensitivity Analysis

The overall effect size regarding the effects of flaxseed oil on GSH levels was sensitive to Soleimani et al. [23]. The study means that with removing this study, the overall results were changed to statistical significance. By removing Raygan et al. [24] (46.12 mmol/L; 95% CI: −0.07, 92.31) and Jamilian et al. [11] (103.93 mmol/L; 95% CI: −11.26, 219.13) studies, the significant effect of flaxseed oil on TAC levels became nonsignificant.

#### 3.3.5. Publication Bias and Trim-and-Fill Analysis

There was no document of publication bias for the influence of flaxseed oil supplementation on GSH (Begg's test; *P*=0.089) (Figure 3(b)) and TAC levels (Begg's test; *P*=0.221) (Figure 4(b)). Moreover, the visual inspection of the funnel plot and Begg's and Egger's tests rejected our hypothesis about the presence of substantial publication bias (*P*=0.711 and 0.562, respectively) (Figure 2(b)).

## 4. Discussion

In this meta-analysis, our results showed that flaxseed oil supplementation significantly decreases MDA serum levels and increases the TAC levels significantly. No significant effect was observed on GSH levels. After subgroup analysis based on administered dosage, sample size, mean age, and duration, the level of heterogeneity was reduced. Flaxseed oil supplementation for ≤8 weeks significantly reduced MDA levels, whereas interventions for >8 weeks did not. In addition, the dose-dependent subgroup analysis showed that a flaxseed oil dosage of ≤2000 mg/day led to a significant decrease in the MDA level. Some leading causes of the observed variation in the findings may be food intake, physical activity, and the participant's clinical state. In the study conducted by Jamilian et al., a 6-week supplementation with 2000 mg/day flaxseed oil in patients with GDM significantly decreased MDA levels [11]. Similar to the previous study, two studies have shown that flaxseed oil significantly reduces MDA [26, 33]. However, there are some studies that showed the effects of 1000 mg/day of flaxseed oil on the MDA level were not significant [23]. One possible explanation could be related to the mean age of the participants (mean age 59). Studies have shown that humans are stronger in middle ages in terms of immune system function and antioxidant status than in old ages and childhood [36]. In total, flaxseed oil is a rich source of alpha-linolenic acid as omega-3 fatty acid and some effective phytoestrogens [12, 13]. Thus, flaxseed oil affects MDA via its significant content of omega-3 and phytochemicals. It has been suggested that flaxseed oil supplementation upregulates the low-density lipoprotein (LDL) receptor and peroxisome proliferator-activated receptor gamma (PPAR-*γ*), reducing malondialdehyde levels by preventing them from being exposed to free radicals [37]. Other mechanisms of the effect of flaxseed oil supplementation on the reduction of malondialdehyde and fatty acid peroxidation are changes in the composition of prostaglandins [7]. Also, some studies have shown that flaxseed oil reduces NF-*κ*B-induced gene expression [38], modification of MAP kinase, AKT signaling pathways [39], and NADPH oxidase activity enhancement [40] which is involved in the stress oxidative pathway. In other words, according to evidence from molecular studies, omega-3 fatty acids affect syntaxin-3 expression. Therefore, omega-3 fatty acids may reduce lipid peroxidation by stimulating syntaxin-3 [41].

Increasing TAC levels via flaxseed oil supplementation was another important finding of this meta-analysis. The mean age of participants and flaxseed type were found to be potential sources of heterogeneity in subgroup analysis. In addition, the subgroup analysis in terms of mean age (>48), dosage, duration, and type of flaxseed products (omega-3 fatty acid from flaxseed oil) did not result in any significant difference. Moreover, in a study on patients with polycystic ovary syndrome [22], 1000 mg/day of flaxseed oil supplementation significantly increased TAC levels, while in another study, 2000 mg of flaxseed oil led to no significant change in TAC levels [11]. The possible mechanism of the effect of flaxseed oil on increasing TAC may be due to decreased ROS production. Also, previous studies have shown that supplementation of flaxseed oil increases J3-isoprostanes which induce NF-E2-related factor 2 (Nrf-2) gene expression [42, 43]. Nrf2, which is involved in the gene expression of antioxidant enzymes, may regulate the detoxification of ROS [43]. In addition to omega-3 fatty acids, the phytochemical content of flaxseed oil, such as lignin, vitamin E, and flavones, has an essential role in amplifying TAC levels [44].

Results obtained from the analysis showed that flaxseed oil supplementation had no significant effect on the GSH level. The mean age of participants was found to be a possible source of heterogeneity in subgroup analyses. The heterogeneity was more considerable in studies with mean age >48. However, GSH levels were significantly increased in participants aged <48 and in the studies that used flaxseed oil-derived omega-3 fatty acids. Previous studies have shown that 2000 mg/day of flaxseed oil supplementation can increase GSH levels [11]. On the contrary, 1000 mg/day of flaxseed oil did not affect GSH levels [23]. The effect of flaxseed supplementation on GSH levels may be due to the possible impact of the omega-3 fatty acid mechanism on the mitochondrial oxidant state and alteration of mitochondrial membrane phospholipid fatty acid composition [45, 46]. In addition, glutathione plays a major role in maintaining a favorable state for mitochondrial oxidation [47].

An earlier meta-analysis [26] showed a significant reduction in serum MDA. It increased TAC, but no change in the GSH level was observed in patients with metabolic syndrome and related disorders following supplementation with flaxseed oil. The results were similar to those of the present study. However, some limitations of a previous meta-analysis [26] may distort its findings. For example, many publications have been missed and not included in the analysis despite being eligible according to inclusion criteria [24, 33, 35]. Moreover, additional analyses such as publication bias, trim-and-fill, and sensitivity analyses had not been performed.

The following are some of the meta-analysis strengths: all of the included articles were RCTs from the last ten years, providing a relevant and up-to-date review. The majority of the included studies had a low risk of bias and were of high methodological quality. Our study also has some limitations: although significant between-study heterogeneity was observed, the potential sources of heterogeneity were revealed to be the differences in the treatment dose and duration. Moreover, the number of studies included in this review was few.

## 5. Conclusions

Overall, the results of this meta-analysis showed that flaxseed oil supplementation reduced MDA levels. Moreover, flaxseed oil supplementation increased the TAC levels but was not effective on GSH levels. It seems that flaxseed oil could be considered as an effective agent in augmenting the antioxidant defense system which indicates the efficiency of being set in the regular diet. However, additional clinical trials with larger sample sizes in various health conditions are required to clarify this more accurately.

## Figures and Tables

**Table 1:** Study characteristics of included studies.

Citation (first author et al., year)	Location	Study population	Sample size (control/intervention)	Mean age (control/intervention)	Intervention/daily dose	Duration (week)	Measured outcomes and results
Pilar et al., 2014	Brazil	MetS	24/20	45–55/45–55	Golden flaxseed/40 g	4	MDA (**↓**)

Mirfatahi et al., 2016	Iran	HD	17/17	59.0 ± 16.4/68.0 ± 12.3	Flaxseed oil/6 g	8	MDA (Ns)

Soleimani et al., 2017	Iran	DN	30/30	62.4 ± 9.6/62.9 ± 10.5	Omega-3 fatty acids from flaxseed oil/1000 mg	12	TAC (**↑**), MDA (Ns), GSH (Ns)

Soleimani et al., 2017	Iran	DFU	30/30	59.9 ± 9.2/58.8 ± 11.2	Omega-3 fatty acids from flaxseed oil/2000 mg	5	TAC (**↑**), MDA (Ns), GSH (**↑**)

Akrami et al., 2017	Iran	MetS	26/26	48.8 ± 6.4/48.3 ± 6.9	Flaxseed oil/25 ml	7	MDA (Ns)

Raygan et al., 2019	Iran	T2DM with CHD	30/30	62.0 ± 13.0/64.6 ± 9.1	Omega‐3 fatty acids from flaxseed oil/2000 mg	12	TAC (**↑**), MDA (**↓**), GSH (**↑**)

Jamilian et al., 2020	Iran	GDM	25/26	28.5 ± 4.1/29.5 ± 5.0	Omega-3 fatty acids from flaxseed oil/2000 mg	6	TAC (Ns), MDA (**↓**), GSH (**↑**)

Rezaei et al., 2020	Iran	NAFLD	34/34	40.8 ± 8.7/45.5 ± 8.7	Flaxseed oil/20 g	12	TAC (Ns), MDA (Ns)

DFU: diabetic foot ulcer; GDM: gestational diabetes mellitus; GSH: glutathione; MDA: malondialdehyde; T2DM: type 2 diabetes mellitus; CHD: coronary heart disease; TAC: total antioxidant capacity; NAFLD: nonalcoholic fatty liver disease.

**Table 2:** Results of risk of bias assessment for randomized clinical trials included in the current meta-analysis on the effects of flaxseed oil supplementation on oxidative stress biomarkers^1^.

Study	Random sequence generation	Allocation concealment	Reporting bias	Other sources of bias	Performance bias	Detection bias	Attrition bias
Pilar et al., 2014	U	U	L	U	H	H	H
Mirfatahi et al., 2016	L	L	H	U	L	H	L
Soleimani et al., 2017	L	L	L	L	L	L	L
Soleimani et al., 2017	L	L	L	L	L	H	L
Akrami et al., 2017	L	L	L	U	U	U	L
Raygan et al., 2019	L	L	L	L	L	L	L
Jamilian et al., 2020	L	L	L	L	L	L	L
Rezaei et al., 2020	L	L	L	U	L	L	L

^1^Each study was assessed for risk of bias using the Cochrane risk of bias assessment tool [21]. Domains of assessment included random sequence generation, allocation concealment, reporting bias, performance bias, detection bias, attrition bias, and other sources of bias. Each domain was scored as “high risk” if it contained methodological flaws that may have affected the results, “low risk” if the flaw was deemed inconsequential, and “unclear risk” if information was insufficient to determine. If a study obtained “low risk” for all domains, it is considered as a high-quality study with totally low risk of bias.

**Table 3:** Pooled estimates of flaxseed oil effects on antioxidant system function within different subgroups.

Variables	No. of studies	WMD or SMD (95% CI)	*P* value	*I*^2^ (%)	*P* heterogeneity
*MDA*					
Total	8	−0.52 (−0.89, −0.15)	0.006	71.3	<0.001
*FL dosage* (mg)					
≤2000	4	−0.84 (−1.11, −0.56)	<0.001	68.0	0.025
>2000	4	−0.14 (−0.43, 0.14)	0.311	—	0.398

*Sample size*					
≤26	4	−0.53 (−0.83, −0.22)	<0.001	82.7	<0.001
>26	4	−0.48 (−0.74, −0.23)	<0.001	57.3	0.071

*Intervention duration (week)*					
≤8	4	−0.53 (−0.83, −0.22)	<0.001	82.7	<0.001
>8	4	−0.48 (−0.74, −0.23)	<0.001	57.3	0.071

*Mean age (years)*					
≤48	4	−0.43 (−0.71, −0.15)	0.002	84.6	<0.001
>48	4	−0.57 (−0.84, −0.29)	<0.001	32.6	0.217

*Type of flaxseed*					
Flaxseed oil	5	−0.33 (−0.58, −0.08)	0.010	62.3	0.031
Omega-3 fatty acid from flaxseed oil	3	−0.78 (−1.10, −0.46)	<0.001	77.5	0.012

*GSH*					
Total	4	0.45 (−0.12, 1.01)	0.122	77.9	0.004
*Mean age (years)*					
≤48	1	1.19 (0.59, 1.79)	<0.001	—	—
>48	3	0.21 (−0.08, 0.51)	0.159	62.3	0.070

*Type of flaxseed*					
Flaxseed oil	1	0.34 (−0.17, 0.85)	0.187	—	—
Omega-3 fatty acid from flaxseed oil	3	0.43 (0.12, 0.74)	0.007	85.2	0.001

*TAC*					
Total	5	82.84 (19.80, 145.87)	0.010	92.7	<0.001
*Mean age (years)*					
≤48	2	−0.02 (−0.03, −0.01)	0.003	66.0	0.086
>48	3	143.4(101.22, 185.61)	<0.001	72.7	0.026

*Type of flaxseed*					
Flaxseed oil	2	−0.02 (−0.03, −0.01)	<0.001	66.0	0.003
Omega-3 fatty acid from flaxseed oil	3	46.88 (19.85, 73.92)	0.023	51.8	<0.001

MDA: malondialdehyde; GSH: glutathione; TAC: total antioxidant capacity; SMD: standardized mean difference; WMD: weighted mean difference; CI: confidence interval.
